# Outcome of adult patients with X-linked hypophosphatemia caused by *PHEX* gene mutations

**DOI:** 10.1007/s10545-018-0147-6

**Published:** 2018-02-19

**Authors:** Douglas Chesher, Michael Oddy, Ulpee Darbar, Parag Sayal, Adrian Casey, Aidan Ryan, Annalisa Sechi, Charlotte Simister, Aoife Waters, Yehani Wedatilake, Robin H. Lachmann, Elaine Murphy

**Affiliations:** 1 Charles Dent Metabolic Unit, National Hospital for Neurology and Neurosurgery, Queen Square, London, WC1N 3BG UK; 2 New South Wales Health Pathology, Royal North Shore Hospital, St Leonards, NSW Australia; 3 Department of Trauma and Orthopaedics, University College London Hospitals NHS Foundation Trust, London, UK; 4 Department of Periodontology, Eastman Dental Hospital, London, UK; 5 Department of Neurosurgery, National Hospital for Neurology and Neurosurgery, Queen Square, London, UK; 6 Chemical Pathology and Metabolic Medicine, University Hospital Southampton NHS Foundation Trust, Southampton, UK; 7 Centre for Rare Disease, Academic Hospital of Udine, Udine, Italy; 8 Institute of Child Health, Great Ormond Street Hospital for Children NHS Foundation Trust, London, UK; 9 Imperial College London NHS Foundation Trust, London, UK

**Keywords:** X-linked hypophosphatemia, XLH, Phosphate regulating endopeptidase homologue, PHEX, Osteotomy, Enthesopathy, Nephrocalcinosis, Dental abcess

## Abstract

X-linked hypophosphatemia (XLH) is the most common monogenic disorder causing hypophosphatemia. This case-note review documents the clinical features and the complications of treatment in 59 adults (19 male, 40 female) with XLH. XLH is associated with a large number of private mutations; 37 different mutations in the *PHEX* gene were identified in this cohort, 14 of which have not been previously reported. Orthopaedic involvement requiring surgical intervention (osteotomy) was frequent. Joint replacement and decompressive laminectomy were observed in those older than 40 years. Dental disease (63%), nephrocalcinosis (42%), and hearing impairment (14%) were also common. The rarity of the disease and the large number of variants make it difficult to discern specific genotype-phenotype relationships. A new treatment, an anti-FGF23 antibody, that may affect the natural history of the disease is currently being investigated in clinical trials.

**Electronic supplementary material:**

The online version of this article (10.1007/s10545-018-0147-6) contains supplementary material, which is available to authorized users.

## Introduction

Hypophosphatemic rickets is a heterogeneous group of inherited disorders characterised by hypophosphatemia and impaired bone mineralisation leading to rickets. The most common inherited form is X-linked hypophosphatemia (XLH) with an estimated prevalence of approximately 4.8 per 100,000 (Beck-Nielsen et al 2009). XLH results from mutations affecting the phosphate regulating endopeptidase homologue gene (*PHEX*) for which more than 300 pathogenic variants have been described (Rowe et al 1996; Sabbagh et al 2000).

*PHEX* codes for a zinc metalloendopeptidase that has been shown to bind to matrix extracellular phosphoglycoproteins (MEPE), and inactivate the inhibitory effect of these proteins on bone mineralisation (Rowe 2012; Addison et al 2008). Through mechanisms that have yet to be fully understood, PHEX activity leads to reduced expression of FGF23 (Rowe 2012). FGF23 interacts with ∝-klotho and FGF receptors to downregulate the expression of the renal phosphate transporters NPT2a and NPT2c and CYP27B1 in the proximal tubules (Bergwitz and Juppner 2010). Inactivating mutations within the *PHEX* gene are associated with increased circulating levels of FGF23 (Bergwitz and Juppner 2010) resulting in phosphaturia, hypophosphatemia, and impaired activation of 25-hydroxyvitamin D to 1,25 dihydroxy-vitamin D.

Clinical manifestations of XLH can vary but most patients will present with limb deformity, primarily affecting the legs, and short stature. Treatment in childhood consists of phosphate and active vitamin D supplementation, with surgical correction of limb alignment if required. Complications from the disease or its treatment include arthritis, enthesopathy (ligament, tendon or joint capsule calcification), dental disease, tertiary hyperparathyroidism, hearing impairment, optic atrophy and nephrocalcinosis (Carpenter et al 2011; Pavone et al 2015). The phenotypic features and complications of XLH are well described by Reid et al (1989), but most of their adult patients were not on active treatment. Beck-Nielsen et al also provided an updated description of the phenotype of XLH in 38 adult patients (Beck-Nielsen et al 2010).

Studies have shown that there is significant morbidity associated with the condition as patients get older, particularly with reduction in mobility, pain and discomfort, caused by osteoarthritis, enthesopathy or insufficiency fractures (Forestier-Zhang et al 2016; Che et al 2016). Current medical treatments, when started very early in childhood (<1 year) can increase final height, but, as biochemical abnormalities persist, and long-term follow up is not yet available, it remains uncertain whether very early treatment can prevent most late-onset adult complications (Quinlan et al 2012). A recombinant monoclonal antibody that will modify the underlying metabolic defect by targeting FGF23 is now in clinical trial (Carpenter et al 2014). It is important therefore to fully understand the natural history of the disease in order to ultimately assess the value of these agents in potentially ameliorating the chronic phenotype of the condition.

This study represents the largest single-centre outcome review of adults with XLH due to confirmed *PHEX* gene mutations and contributes to the available natural history data of the condition.

## Patients and methods

The clinical records of 59 patients with XLH attending a single inherited metabolic disease service from 1998 were retrospectively reviewed. Data were retrieved from both electronic and paper patient records and were reviewed for the presence of the clinical features and complications of familial hypophosphatemia including bone disease, renal disease, dental disease, spinal disease and other features such as hearing loss. A history of neoplastic disease was also recorded when present. A mean systolic and diastolic blood pressure (BP) was calculated from the three most recently recorded measurements.

The most recent concentration or activity of haemoglobin, parathyroid hormone (PTH), alkaline phosphate (ALP), calcium, albumin, creatinine, phosphate and urine calcium:creatinine ratio was recorded. Routine chemistry tests were assayed using a Roche Cobas 8000 Modular Analyser using the manufacturers standard reagents. Vitamin D was measured by liquid chromatography tandem mass spectrometry. Haemoglobin was measured as part of the full blood count on a Sysmex XE series automated haematology analyser. The significance of biochemical and haematological analysis was assessed by comparing results to laboratory supplied reference intervals for adults. Estimated glomerular filtration rate (eGFR) was calculated using the Chronic Kidney Disease Epidemiology Collaboration (CKD-EPI) equation (Levey et al 2009) using the patients most recent serum creatinine concentration, age at last visit and gender. Urine phosphate measurements were not routinely performed as part of regular clinical management of patients, so the ratio of tubular transport maximum reabsorption of phosphate to glomerular filtration rate (TmP/GFR) could not be calculated.

*PHEX* gene mutation analysis was performed by a local reference laboratory (Exeter, UK) using a combination of polymerase chain reaction (PCR) with direct Sanger sequencing and Multiplex Ligation-dependent Probe Amplification (MLPA), MRC Holland kit P223.

When available, results of ultrasound imaging of the renal tract detailing any evidence of nephrocalcinosis, renal calculi, scarring or atrophy were recorded. Available plain film skeletal radiographs, dental imaging, computerised tomography and magnetic resonance images were reviewed by a single orthopaedic, dental or spinal surgeon as appropriate. The treatment regimen of all patients was recorded, and prescribed drugs were categorised regardless of dose.

## Statistical analysis

Statistical analysis was performed using the R statistical package version 3.2.4 (R-Core 2016). The Shapiro-Wilk test was used to assess for normality. Non-normally distributed variables were expressed as medians and associated interquartile ranges. Associations between categorical variables were assessed with a Chi square test with Yates continuity correction. Linear correlation between continuous variables was assessed using Pearson’s correlation coefficient.

## Results

The study group consisted of 19 males and 40 females, with a median age at last visit of 37 years, ranging from 17 to 79 years. Patient characteristics, biochemical test results and medications are shown in Table 1.

**Table 1:** Patient characteristics

Number	Male	Female	All
	19	40	59
Age (years) [range]	36 [22–79]	38 [17–74]	37 [17–79]
Height (cm) [UK mean height (Moody 2012)]	162 (158–168) [175.3 cm]	153 (146–156) [161.9 cm]	155 (149–161)
*Ethnicity*			
White-British	17	35	52
Black-British	2	3	5
Other	0	2	2
Body mass index (kg/m^2^)	27.6 (24.8–33.7)	25.3 (23.5–30.2)	26.8 (23.9–30.7)
Mean systolic blood pressure (mmHg)	130 (121–142)	120 (110–127)	122 (113–135)
Mean diastolic blood pressure (mmHg)	81 (73–88)	75 (71–80)	76 (71–81)
*Medications*			
Antihypertensive	5	11	16 (27%)
Lipid lowering	2	1	3 (5%)
Antidepressant	0	3	3 (5%)
Regular anti-inflammatory	3	4	7 (12%)
*Treatment at last consultation*	Taking vitamin D	NOT taking vitamin D	All
Males	15	4	19
Females	25	15	40
Haemoglobin (RI: 130–170 g/L)	144 (135–153)	139 (132–157)	143 (133–152)
Parathyroid hormone (RI: 1.6–6.9 pmol/L)	6.4 (4.6–7.8)	6.9 (4.8–7.4)	6.6 (4.7–8.0)
Alkaline phosphatase (RI: 40–129 U/L)	104 (86–124)	97 (69 – 115)	101 (80–121)
Albumin adjusted calcium (RI: 2.20–2.60 mmol/L)	2.35 (2.28–2.46)	2.34 (2.29–2.39)	2.34 (2.29–2.42)
Serum phosphate (RI: 0.87–1.45 mmol/L)	0.64 (0.58–0.73)	0.62 (0.58–0.70)	0.63 (0.58–0.72)
Serum creatinine (RI: 66–112 umol/L)	65 (55–73)	65 (58–71)	65 (57–72)
Estimated GFR (mL/min/1.73m^2^)	113 (94–124)	107 (89–116)	110 (90–124)
Urine calcium/creatinine ratio (RI: 0.08–0.79 mmol/mmol)	0.27 (0.20–0.54)	0.15 (0.07–0.40)	0.23 (0.15–0.47)
Vitamin D (μg/day) – dose at last consultation	0.5 (0.5–1.25)	–	–
Phosphate supplementation (mg /day) – dose at last consultation	500 (470–1000)	–	–

Values are reported as medians (interquartile range) unless otherwise specified

Mutation analysis of the *PHEX* gene was directly performed in 48 patients, from 35 kindreds. In a further 11 patients the genotype could be inferred from the result of a first degree relative. The 37 distinct mutations identified were private to individuals or known family groups and are summarised in Table 2. Of these, to the best of our knowledge, 14 mutations have not previously been reported.

**Table 2:** *PHEX* gene mutations identified in this cohort

Location	cDNA (NM_000444.5)^a^	Protein	Mutation type	PHEXdb^b^ (if assigned)
Intron 3	c.350-2A > G	NA	splicing	
Intron 3	c.350-1G > T	NA	splicing	218
Intron 4	c.436 + 1G > T	NA	splicing	NRB
Exon 5	c.503C > T	p.P168L	missense	
Exon 5	c.542C > G	p.S181X	nonsense	NRB
Exon 5	c.630del T	p.Asp211fs	frameshift	NRB
Intron 6	c.732 + 1G > C	NA	splicing	NRB
Exon 8	c.850delA	NA	frameshift	
Exon 8	c.871C > T	p.R291X	nonsense	6
Exon 9	c.1033C > T	p.Q345X	nonsense	
Exon 10	c.1158G > A	p.W386X	nonsense	120
Intron 10	c.1173 + 1G > T	NA	splicing	273
Exon 11	c.1217G > T^1^	p.C406F	missense	NRB
Exon 12	c.1303-?_1404 +?del	NA	deletion	28
Exon 12	c.1357_1360del	p.E453fs	frameshift	NRB
Exon 12	c.1366 T > A^2^	p.W456R	missense	NRB
Exon 12	c.1368G > C	p.W456C	missense	
Exon 13	c.1474del	p.L492 fs	frameshift	NRB
Intron 13	c.1482 + 5G > C	NA	splicing	121
Exon 14,15	c.1483-?_1645 +?del	NA	deletion	NRB
Exon 15	c.1600C > T^3^	p.P534S	missense	NRB
Exon 15	c.1601C > T	p.P534L	missense	71
Exon 15	c.1645C > T	p.R549X	nonsense	13
Exon 16	c.1646_1700 del	NA	deletion	
Exon 16	c.1699C > T	p.R567X	nonsense	236
Exon 16	c.1670del	p.K557 fs	frameshift	NRB
Exon 17	c.1735G > A	p.G579R	missense	90
Exon 17	c.1739A > C	p.H580P	missense	
Exon 18	c.1775_1778dupAATA	NA	frameshift	
Exon 18	c.1830delG	p.K610NfsX7	frameshift	
Exon 18	c.1874_1875dupAT	pY626IfsX8	frameshift	NRB
Exon 19	c.1958C > A	p.A653D	missense	310
Intron 19	c.1965 + 1G > A	NA	splicing	62
Exon 20	c.1978_1988del11	p.Trp660fs	frameshift	NRB
Exon 20	c.2018 T > G^4^	p.L673R	missense	NRB
Exon 20	c.2066C > A	p.A689D	missense	

NA: Not applicable

^a^NCBI reference sequence

^b^PHEXdb mutation ID (if assigned)

NRB: Not reported before (new mutation)

^1,2,3,4^Missense substitutions. All highly conserved amino acids, up to *C. elegans* (considering 12 species). MutationTaster: disease causing (Schwarz et al 2014). ^1,2,4^SIFT: deleterious. ^3^SIFT: tolerated (Kumar et al 2009)

### Orthopaedic/bone complications

**Fig. 1:**
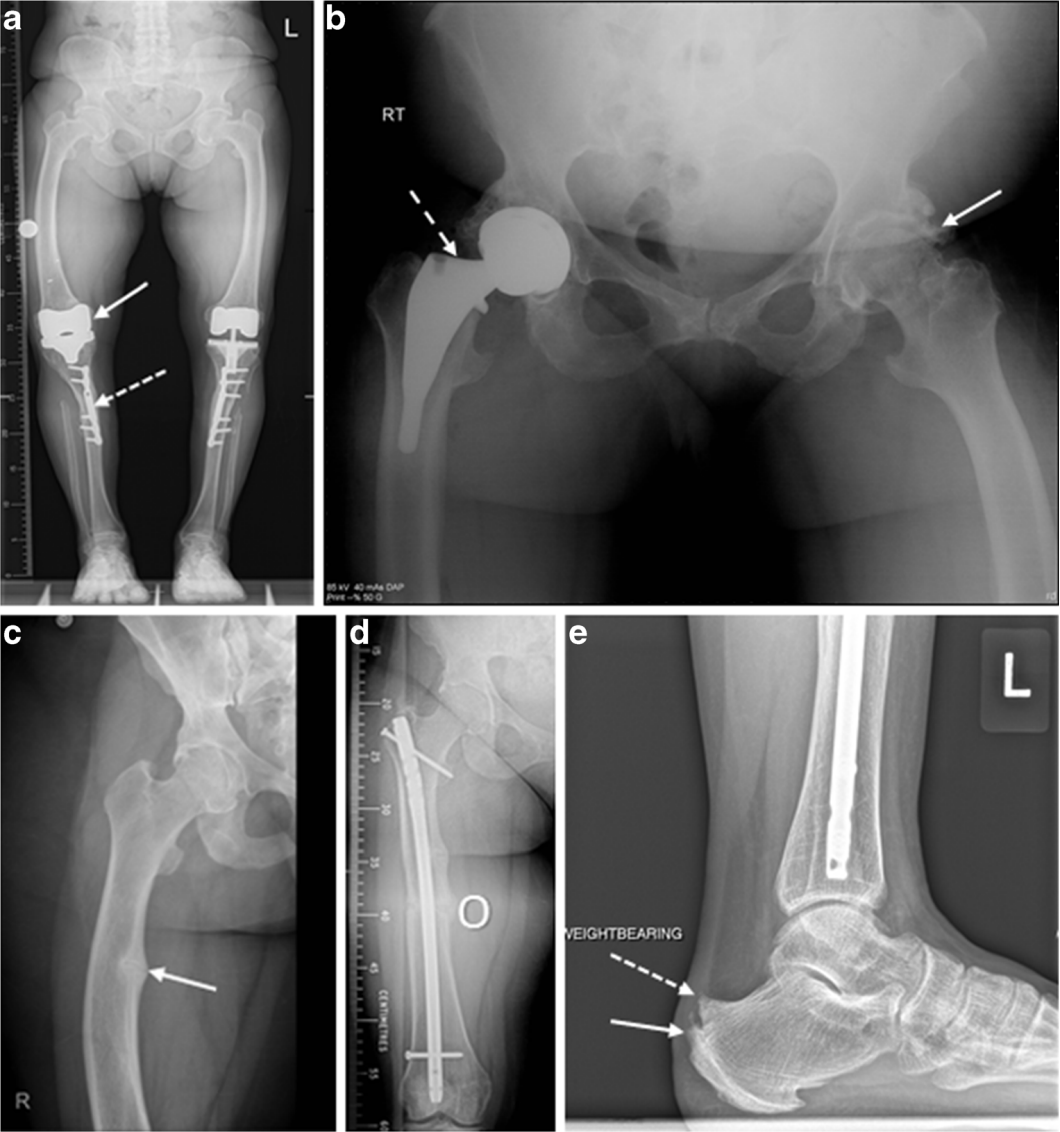
Orthopaedic complications of XLH. **a** Standing antero-posterior limb alignment radiograph of a 75 years old female demonstrating bilateral bowed coxa vara deformity of the proximal femur with hip osteoarthritis, femoral shaft bowing, bilateral total knee replacements (solid arrow), tibial shaft plates from osteotomies in adolescence (dashed arrow) and ankle arthritis with loss of joint space. **b** Antero-posterior pelvic radiograph of a 60 years old female demonstrating left hip osteoarthritis showing loss of joint space and osteophytes (solid arrow) and a right short stem un-cemented total hip replacement (dashed arrow). **c-d** Antero-posterior radiograph of the right femur of a 33 years old female with a Looser’s zone of the medial cortex of the femoral shaft (solid arrow) treated with a femoral shaft osteotomy (O) and intra-medullary rod fixation to realign the bone shape and aid fracture healing. **e** Lateral standing ankle radiograph of a 32 years old female showing a prominent posterior/superior corner Haglund’s deformity of the os calcis (dashed arrow) and insertional Achilles’ tendon bony spur enthesophytes (solid arrow)

Skeletal manifestations were very common (Table 3, Fig. 1a–e). As expected, both men and women were of short stature (Table 1). All patients remained ambulant, none were regular wheelchair users. Symptomatic or progressive deformity was the most common lower limb manifestation of the disease with 27 patients having undergone long bone osteotomies or guided growth surgery with physeal stapling. These had been undertaken in various combinations of unilateral, bilateral or combined tibial and femoral procedures through childhood and adulthood. There were no significant associations between a previous history of osteotomy and factors including the presence of pseudofractures on imaging, the degree of secondary hyperparathyroidism (most recent PTH level) and current treatment with vitamin D and phosphate (data not shown). Symptomatic hip and knee joint disease was present in 19 patients ranging from focal osteochondral defects to end stage osteoarthritis. Three patients had undergone a total of five cemented total knee replacements (two bilateral) and two patients had undergone unilateral un-cemented total hip replacements with all arthroplasty operations having been undertaken at greater than 48 years of age. Seven patients developed symptomatic degenerative ankle or foot joint disease with three having undergone operative microfracture treatment for osteochondral defects or resection of symptomatic osteophytes for bony impingement. Six patients developed insertional Achilles’ tendinopathy with symptomatic enthesophytes. These were all managed non-operatively with orthoses and physical therapy and none required invasive treatments.

**Table 3:** XLH complications, including surgical procedures in 59 adult patients

Procedure/complication	Male	Female	All (%)
*Lower limb involvement*			
Osteotomy - tibial unilateral	–	4	4 (7)
Osteotomy - tibial bilateral	6	3	9 (15)
Osteotomy - femoral unilateral	–	5	5 (8)
Osteotomy - femoral bilateral	1	2	3 (5)
Osteotomy - femoral and tibial bilateral	3	1	4 (7)
Total number of individuals with at least one osteotomy	10	15	25 (42)
Guided growth/physeal stapling	2	2	4 (7)
Knee osteochondritis dissecans/osteoarthritis	2	5	7 (12)
Hip osteoarthritis +/- coxa vara	3	9	12 (20)
Total knee replacement	–	1	1 (2)
Bilateral total knee replacement	1	1	2 (3)
Total hip replacement	–	2	2 (3)
Ankle achilles enthesopathy (non-operative)	3	3	6 (10)
Ankle arthritis/osteochondritis dissecans talus	2	5	7 (12)
*Spinal involvement*			
Ossification of the ligamentum flavum	2	3	5/7
Ossification of the posterior longitudinal ligament	3	1	4/7
Laminectomy/fixation – thoracic	1	2	3 (5)
Laminectomy/fixation – lumbar	0	1	1 (2)
Cervical discectomy and fusion	0	1	1 (2)
Cervical disc prolapse	0	1	1 (2)
Chiari malformation	3	0	3 (5)
Spinal cord syrinx	1	0	1 (2)
*Other complications*			
Any dental disease	15	22	37 (63)
Nephrocalcinosis	5	11	16/38 (42)
Hearing impairment	4	4	8 (14)
Parathyroidectomy	1	2	3 (5)

### Spinal complications

Seven patients were investigated, clinically and radiologically, for symptoms attributable to the spine (Table 3).

**Fig. 2:**
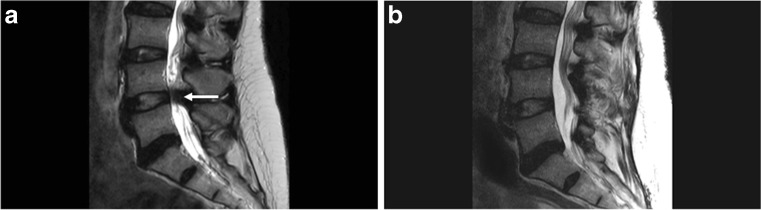
Spinal complications of XLH. **a** MRI lumbar spine showing significant canal stenosis at L4–5 secondary to ossification of the ligamentum flavum. **b** Post-operative MRI lumbar spine of the same patient showing significant improvement in the dimensions of the canal after lumbar laminectomy

A 55 year old female presented with neurogenic claudication due to severe lumbar canal stenosis secondary to an ossified ligamentum flavum (OLF) (Fig. 2a). She underwent a lumbar laminectomy with significant post-operative radiological and clinical improvement. On 2 year follow-up however, new mild stenosis has been noted at the proximal lumbar levels (Fig. 2b).

The other six patients (four male, two female) had radiological cervical and/or thoracic cord compression, presenting at age 43 to 67 years. Three of them were managed conservatively with radiological and clinical surveillance as they had minimal symptoms/signs in spite of moderate to significant cord compression. One of the conservatively managed patients has moderate cervical cord compression due to ossification of the posterior longitudinal ligament (OPLL), another has mild compression at T9/10 and T10 due to OLF, and the third has a Chiari malformation, a syrinx and OPLL (Suppl. Fig. 1a-b).

Three patients with cord compression had significant myelopathy and underwent surgical interventions, all with good outcomes. One of these patients initially underwent thoracic laminectomy for myelopathy and re-presented 10 years later with cord compression proximal and distal to the previous decompression (Suppl. Fig. 2a-b). This was treated by extending the decompression/laminectomies augmented with pedicle screw fixation (Suppl. Fig. 2c-d).

**Fig. 3:**
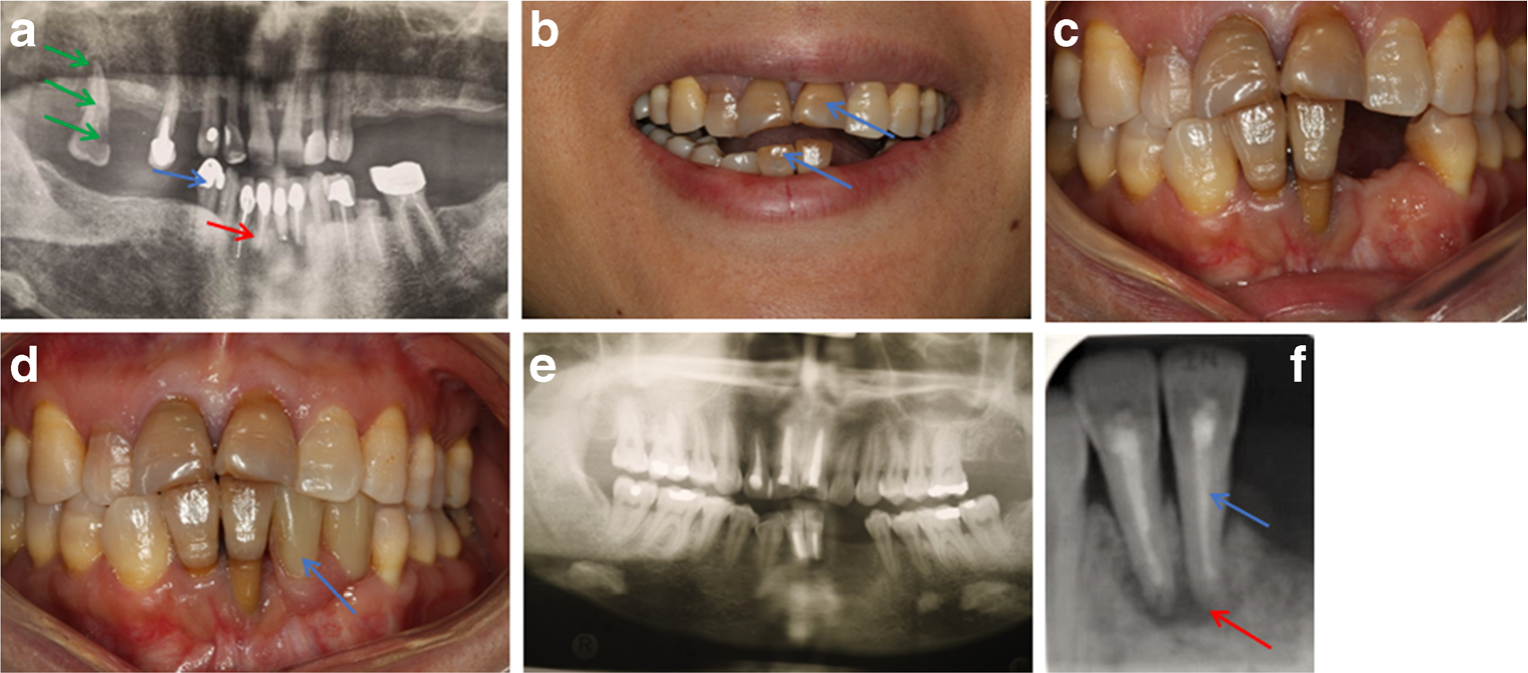
Dental complications of XLH. **a** Radiograph of a patient showing taurodont molars. Large pulp chambers are evident with an abscess on the upper right molar and large root canals (green arrows). Multiple teeth have been root filled (red arrow) and crowned (blue arrow). **b** Patient presenting with recurring abscesses associated with the lower incisor teeth. Note the discolouration (blue arrows) of the lower and upper incisor teeth (the 2 middle teeth). **c** Intraoral view showing the teeth; this patient had presented with unstable periodontal (gum) disease and caries (decay) which has now been treated and 2 teeth have been lost. **d** Patient wearing a lower denture (blue arrow) to replace the missing lower left incisors. **e-f** Dental pantomogram showing root fillings (blue arrow) in the upper right (UR) 2, 1 and upper left (UL) 1 and the lower right (LR) 1, 2 with wide canals and missing lower left (LL) 1, 2. The periapical view of the LR 1, 2 shows the apical infection and the unusual appearance of the bone (red arrow)

Another patient initially presented with cervical myelopathy secondary to a calcified disc and underwent a C6/7 anterior cervical discectomy and fusion. She then re-presented 10 years later with thoracic myelopathy due to multi-level thoracic cord compression with OPLL and OLF (Suppl. Fig. 3a-b). She underwent a thoracic laminectomy which was complicated by a cerebrospinal fluid (CSF) leak which was repaired. She did well neurologically but developed a pseudo-meningocele which was initially managed conservatively but enlarged over several years to become uncomfortable (Fig. 3c). She also developed proximal level thoracic stenosis which eventually needed further decompression and pseudo-meningocele repair (Fig. 3d). Her cervical OPLL has also progressed over several years to cause moderate cord compression, which currently remains asymptomatic.

The four patients requiring spinal surgery were all older than 40 years. Two of these patients were from the same family having a frameshift mutation in exon 13 (c.1474del). The other two patients were from a separate kindred with a deletion in exon 16 (c.1646–1.1kb_1700 + 0.3kb del).

### Dental complications

Dental disease was very common with 37 (63%) patients having at least one form of dental disease, with many having multiple problems with caries, periodontal disease and failing crowns and restorations and missing teeth being the most common. Twenty four (41%) had a history of dental abscess de novo or associated with teeth that had been root filled and 29 (49%) required at least one dental extraction. There was no association between the history of dental abscess or dental extraction with age or current treatment with vitamin D. Figure 3a shows a typical presentation of one of these patients who has had early treatment with vitamin D and phosphate but has undergone root canal treatment and crown placement on many teeth, with some having been lost. Figure 3b–e shows a patient with extensive problems who ultimately lost the lower central incisors and could no longer tolerate a denture. The teeth were then successfully replaced with dental implants.

### Other findings

Thirty eight patients had at least one renal ultrasound performed, and nephrocalcinosis was documented in 16 (42%) of these patients. None of these patients had a history of renal calculi. Age was associated with a progressive decline in eGFR (Suppl. Fig. 4). Two patients had an estimated eGFR <60 mL/min/173m^2^ and one patient had a severely decreased eGFR <25 mL/min/173m^2^.

Hearing impairment was documented by formal audiometry in eight patients (four male, four female) with the prevalence increasing with age. Hearing loss, either symmetric or asymmetric, was predominantly sensorineural in nature, but three patients also had a conductive component.

Neoplastic disease occurred in five patients (one male, four female). These included melanoma of the parotid gland, breast carcinoma and colorectal carcinoma (same patient), Hodgkins lymphoma, phaeochromocytoma (in a patient with an *SDHD* mutation) and a pituitary macroprolactinoma.

Three patients (two females aged 23 years and 16 years, one male aged 69 years) have had successful parathyroid gland removal (three gland removal, total parathyroidectomy and single gland adenoma removal, respectively) for hyperparathyroidism associated with persistent hypercalcemia.

One 19 year old male was noted to have mild asymptomatic papilloedema on routine eye examination. MRI imaging revealed a type 1 Chiari malformation with moderate hydrocephalus. Management has been conservative, with regular monitoring and he has maintained stable visual function, with stable visual fields, normal colour vision and intraocular pressures during 8 years of follow up.

Another 35 year old male patient has severe systemic disease which includes stable chronic renal failure with secondary hypertension and hyperparathyroidism (eGFR 21 ml/min/1.73m^2^, PTH 67 pmol/L (RI:1.6–6.9)), bilateral optic atrophy with significant visual impairment in the left eye and bilateral moderate to severe sensorineural hearing loss. MRI shows diffuse hyperostosis of the calvarium. Hearing impairment was evident from childhood, a right hearing aid being recommended at 14 years. He also has a history of recurrent dental abcesses. Spinal imaging shows diffuse sclerosis of the thoracic and lumbar vertebrae. He has never required an osteotomy or any other orthopaedic procedure. He attained a final height of 158 cm. He was treated from childhood with vitamin D and phosphate until age 30 years. Phosphate supplementation was stopped at 30 years (phosphate levels had normalised with worsening renal impairment). Vitamin D supplementation was stopped at 35 years. This man is from the same kindred as two female patients who have had spinal cord involvement requiring decompression, and thus the phenotype within this family appears severe.

### Pregnancy

Twenty six (76%) children were delivered by caesarean section, with only eight delivered vaginally, of a total of 34 deliveries in 18 women with XLH. This caesarean section rate is much higher than that of the general UK population, in whom 72.9% of deliveries are vaginal and 27.1% by caesarean section (http://www.content.digital.nhs.uk/maternityandchildren/maternityreports).

### Treatment

Fourty one patients were treated with calcitriol (38) or alfacalcidol (2). All patients treated with active vitamin D also received phosphate supplementation. No patient in this cohort was treated with phosphate supplementation alone or cinacalcet. There was no difference in mean parathyroid hormone, alkaline phosphatase, albumin adjusted calcium or phosphate concentration in those on or off treatment with vitamin D. Treatment with vitamin D was associated with a small increase in urine calcium:creatinine ratio in those who were (0.27 mmol/mmol) versus those who were not (0.15 mmol/mmol) on treatment (*p* = 0.02). However, there was no association between the presence or absence of nephrocalcinosis and whether or not the patient was currently being treated with vitamin D.

## Discussion

XLH is the most common form of familial hypophosphatemia. These 59 adult patients exhibited all the features and complications described for those with XLH affecting the skeleton and spine, including dental disease, nephrocalcinosis and renal impairment, sensorineural hearing loss, and hyperparathyroidism requiring parathyroidectomy (Carpenter et al 2011; Pavone et al 2015; Reid et al 1989). A history of tibial and/or femoral osteotomy was common at all ages but spinal stenosis requiring decompression or joint disease requiring knee or hip replacement was only seen in those older than 40 years of age.

Molecular genetic analysis in this series of patients was consistent with previous reports, in that there were a wide variety of mutations that were private to individuals or known kindreds (Gaucher et al 2009). Other studies have not been able to demonstrate a genotype-phenotype relationship in XLH secondary to mutations in the *PHEX* gene (Gaucher et al 2009). Similarly, we have not been able to discern any clear genotype-phenotype correlations in our patient cohort.

Osteotomy was performed in 42% of our patients, which is similar to previous reports suggesting that 24 to 65% of patients will require surgical intervention for lower limb deformity resulting from XLH, despite optimal medical treatment (Horn et al 2017; Evans et al 1980). More recently, with earlier and better medical care, not all patients develop severe deformity. This, combined with the development of guided growth techniques, suggests that extensive surgery and its associated risks (including recurrent deformity and non-union) may be avoidable in future in early diagnosed, optimally treated patients (Quinlan et al 2012; Makitie et al 2003; Carpenter et al 2011; Horn et al 2017). Similarly, restoring normal joint alignment at the knee diminishes the abnormal strain placed on the ligaments in a varus or a valgus knee, and may decrease the incidence of early arthritis, and subsequent need for joint replacement, associated with overt lower limb malalignment (Khan et al 2008).

The medical literature regarding the spinal manifestations of XLH and their surgical management and outcomes is restricted to case reports and short case series (Hirao et al 2016; Soehle and Casey 2002). Ossification of the paraspinal ligaments in XLH is well recognised and the published series document thickening of the laminae, calcification of the discs, facet joint hypertrophy and ossification of the ligamentum flavum and/or posterior longitudinal ligament (Hirao et al 2016; Soehle and Casey 2002; Dunlop and Stirling 1996; Velan et al 2001). These can result in spinal canal stenosis impinging on the spinal cord leading to myelopathy or on the lumbar nerve roots manifesting as neurogenic claudication/radiculopathy. By comparison of lumbar radiographs, Cartwright et al reported narrower spinal canals in XLH patients compared to normal individuals (Cartwright et al 1981). This contributes to their propensity for canal stenosis.

These spinal manifestations have been reported in the setting of both untreated and treated XLH. Concurrent multiple level canal stenosis has also been reported (Kawaguchi et al 2009).

To date however, only 23 cases of XLH undergoing spinal surgery for myelopathy have been reported (Hirao et al 2016).

In our experience synchronous or metachronous involvement of the cervical, thoracic and lumbar spine is not uncommon in treated XLH patients. After surgical decompression, new stenosis at other levels within the thoracic and lumbar spine was also noted. In keeping with the reported literature, our surgically decompressed patients had satisfactory neurological recovery. However, surgery can be challenging particularly in the setting of ossification of the ligamentum flavum and posterior longitudinal ligaments, where CSF leak is not uncommon. Recently, a minimally invasive technique for multi-level thoracic cord decompression has been reported but surgical experience remains limited (Riccio et al 2016).

Another challenging area is decision-making in the setting of significant radiological spinal cord compression without cord signal change in an otherwise asymptomatic patient. We usually opt to closely monitor these patients clinically and radiologically and offer surgical intervention if they develop clear signs or symptoms of cord compression. In asymptomatic patients with cord compression but with definite cord signal change on MRI, we usually opt to offer surgical intervention.

The *PHEX* gene is expressed in osteoblasts, osteocytes and odontoblasts and the genetic mutations associated with XLH lead to dental anomalies and symptomatic dental disease. Dental disease is common with a predominance of papers reporting the formation of spontaneous dental abscesses in the absence of any caries associated with the teeth. These abscesses may often be the first sign of the disease (Seow 1984). Longitudinal studies have shown that XLH does not cause significant changes in rates of dental development; however, dental taurodontism and ectopic permanent canines are often a common feature along with high pulp horns, large pulp chambers and dentinal clefts (Seow et al 1995; Souza et al 2013). It is these clefts that enable bacterial ingress to the pulp causing the dental abscesses in the absence of caries. Histologically, tubular dentinal defects extending from the pulp to the enamel and marked globular dentine which impairs calcification lead to the formation of calcospherites (incompletely mineralised dentine) which trap microorganisms into the tubules. Enamel and dentine formation occurs between 4 months in utero and 11 months after birth, so the effects of dysplastic and poorly mineralised dentine are more widely seen in the primary dentition (Ribeiro et al 2015). Although it is suggested that the dental manifestations can be minimised in the permanent dentition if treatment is started soon after birth, complications, including recurrent abscesses, may still occur as the patient grows older (Batra et al 2006). Our data did not show any difference in rates of dental abscess in those on treatment with vitamin D compared to those not on treatment. However, our records only allowed us to consider treatment when the patient was last reviewed and not lifetime exposure to vitamin D supplements. Other authors have examined this and found that both total lifetime exposure, and exposure in adulthood to treatment with vitamin D and phosphate, were associated with a lower risk of severe dental disease such as recurrent abcesses in adults with XLH (Connor et al 2015). Recently, it has been shown in a human dental pulp cell model of extracellular mineralisation that there is likely also a role for impaired local intrinsic PHEX activity, as well as systemic hypophosphatemia, in the defective mineralisation of individuals with XLH (Coyac et al 2017).

In our cohort of patients, a history of dental abscesses was frequent with more than 60% suffering from dental disease, the majority of whom gave a history of having suffered an abscess at some point. Papers that report dental findings usually focus on children or young patients (<20 years old) with very few having reported what happens to the dentition as these patients get older. Rabbani et al in their cross-sectional study with a mean age of 10 +/− 4.23 years reported that dental decay and delay in the dentition were the most prevalent manifestations followed by enamel hypoplasia. This is at variance to the findings in other studies reported earlier which state that delay in the dentition was not a common feature (Souza et al 2013; Rabbani et al 2012). Our group of patients were adults with a mean age of 37 years and at least one third of them had undergone root canal treatment due to dental abscess formation and other forms of treatment which included the provision of crowns and fillings to protect the teeth due to the problems associated with poorly mineralised dentine.

Treatment options for adults are limited due to the compromised nature of the teeth and a significant majority will give a history of recurring loss of teeth over time (Lee et al 2017). Although earlier tooth removal has been advocated, this is challenging as these patients may also have alveolar atrophy which effects the provision of replacement teeth. Dental implants have become an option; however, due to the mineralisation changes and effects within the alveolar bone, the mechanisms of which remain unclear, outcomes remain unpredictable with very little published in the literature. Age is also a known risk factor for periodontal disease, and this along with the compromised tooth surfaces with hypoplastic and thin enamel places these patients at a higher risk of periodontal infection. Early dental intervention at a young age and regular life-long dental care are essential to reduce the risk of recurrent dental abscesses and tooth loss, which may be inevitable in many of these patients.

We also observed a high rate of nephrocalcinosis with renal ultrasound and a progressive decline in estimated GFR with age. Nevertheless, only one male patient, with severe XLH, had severe chronic renal impairment. GFR is known to decrease with age and a significant proportion of the population greater than 70 years of age will have an estimated GFR less than 60 mL/min/1.73m^2^ (Delanaye et al 2012; Mathew et al 2007). Overall, the decline we observed in estimated GFR therefore appears consistent with the fall in estimated GFR seen in the general population (Mathew et al 2007).

Five of our patients had a history of neoplastic disease, although no particular neoplasm appeared to be over-represented. While XLH has not previously been associated with the development of neoplastic disease, it has been observed that a higher plasma FGF23 concentration is associated with an increased risk of metachronous colorectal neoplasia (Jacobs et al 2011). The authors suggest this might be related to reduced circulating levels of 1,25 dihydroxy vitamin D but they did not report any association between the serum concentration of 1,25 dihydroxy vitamin D and risk of cancer (Jacobs et al 2011). Neither FGF23, nor 1,25 dihydroxy vitamin D levels were routinely measured in our patient cohort and the expected number of neoplasms in our relatively young population of individuals is uncertain, so it is not possible to establish whether this is a significant association or a chance finding.

The prevalence of hearing loss among individuals with XLH is uncertain and is not mentioned in several case series of adults with XLH (Stickler and Morgenstern 1989; Beck-Nielsen et al 2010; Reid et al 1989). Sensorineural hearing loss, often asymmetric, appears most prominent, but a mixed picture, thought to be caused by endolymphatic hydrops is also seen (O’Malley et al 1985). Similar to others, we have found that some individuals with XLH have generalised osteosclerosis and thickening of the petrous bone (O’Malley et al 1988). Due to missing data on treatment in early childhood, we could not examine the impact of treatment with phosphate and vitamin D on hearing. A recent report suggests however that, despite treatment leading to improvement in mineralisation of the otic capsule in the Phex mouse model, there is no impact on endolymphatic hydrops, hearing or vestibular dysfunction (Wick et al 2017).

There are no published case series on pregnancy, labour and delivery in women with XLH. Our policy is to continue women who are stable on active treatment (vitamin D and phosphate) on therapy throughout pregnancy, with monitoring of serum calcium and urinary calcium:creatinine ratios in order to modify treatment accordingly. Those individuals who are not on therapy at the time of conception are generally not started on treatment during pregnancy. We have noted a very high rate of cesaerean section (75%) among our patients, similar to that reported previously (68%) (Reid et al 1989), but considerably higher than that of other studies (14–34%) (Beck-Nielsen et al 2010; Stickler and Morgenstern 1989). The reasons were varied and included breech presentation of the fetus, but most frequently reflected the choice of obstetricians and women with the condition to opt for an elective caesarean section due to the potential complications of delivery associated with short maternal stature, and concerns over a narrowed birth canal and the potential risk of obstructed labour.

Some controversy exists about if and when to treat adult patients with XLH (Carpenter et al 2011). Conventional treatment in adults, if continued, involves the use of activated vitamin D (calcitriol or alfacalcidol) and phosphate, and requires careful monitoring as it may be associated with hypercalcemia, hypercalciuria, hyperparathyroidism, nephrocalcinosis, nephrolithiasis and potentially chronic kidney disease. Forty one of these 59 adults were on treatment for indications including skeletal pain, biochemical evidence of osteomalacia (increased alkaline phosphatase) and around the perioperative period for orthopaedic procedures. In addition, all patients with a history of pseudofractures were on treatment. We observed a higher urinary calcium excretion in those treated with vitamin D. However, there was no association between current treatment with vitamin D and the presence of nephrocalcinosis.

KRN23, a recombinant antibody targeting FGF23, is a novel agent which is under investigation for XLH. To date, it has been shown to increase the renal tubular threshold for phosphate reabsorption, serum phosphate and 1,25(OH)2D3 levels (Carpenter et al 2014; Imel et al 2015). Phase II studies in adults are ongoing (https://clinicaltrials.gov/: NCT02312687, NCT02526160 and NCT02537431).

This study had a number of weaknesses. Firstly, it was highly dependent on observations being recorded by the treating clinicians. Some features may have been considered less important, or unrelated, and therefore less likely to be documented in the patient’s medical record. Others, for example Chiari malformation, were not systematically sought by imaging. Formal hearing assessment was only performed in those who reported subjective problems. Secondly, the service was an adult metabolic unit and access to childhood records was limited, and thus effects of treatment in childhood on the development of complications in adulthood could not be fully addressed. Thirdly, there is no direct comparison to a control group making it difficult to assess whether the frequency of features such as malignancy are significantly different to that expected in the general population.

## Conclusion

Our patients attending a single centre for adults with inherited metabolic diseases presented with all the features commonly associated with XLH. The bone manifestations of the disease are common and frequently require corrective surgery. Decompressive spinal surgery and joint replacement were indicated in several individuals older than 40 years of age. Patients need regular dental review and education in order to try and reduce the high frequency of dental disease. While there was a high rate of nephrocalcinosis, the progressive decline in renal function appears similar to that observed in the general population, and only one patient had severe chronic renal disease.

Currently available treatments for XLH do not appear to fully address the long-term complications of the condition, which is associated with considerable morbidity in adulthood. Long-term, comprehensive ongoing data collection will be needed to enable physicians to determine with some certainty, whether the use of novel agents, in addition to the expected improvement of the biochemical abnormalities, can also prevent the development of complications still seen despite existing treatments of XLH.

## Electronic supplementary material

Suppl. Fig. 1 a MRI cervical spine showing a Chiari malformation (dotted arrow), a syrinx at C6,C7 (dashed arrow) and spinal cord compression at C5–6 (solid arrow) secondary to ossification of the posterior spinal ligament. b CT cervical spine, an axial image showing significant reduction in the canal diameter due to ossification of the spinal ligament (solid arrow) (GIF 517 kb)

High resolution image (TIFF 618 kb)

Suppl. Fig. 2 a MRI thoracic spine showing previous thoracic laminectomy and new spinal cord compression above and below the decompression secondary to ossification of the ligamentum flavum. b CT thoracic spine axial image showing ossification of the ligamentum flavum causing canal stenosis. c Post-operative MRI thoracic spine showing well-decompressed spinal cord after thoracic laminectomy and fixation (the wavy cord appearance is due to metal artefact). d Post-operative CT thoracic spine showing absence/removal of the ossified ligamentum flavum after laminectomy and screw fixation (GIF 459 kb)

High resolution image (TIFF 549 kb)

Suppl. Fig. 3 a MRI cervical spine showing ossification of the posterior longitudinal ligament (solid arrow) causing minor canal stenosis. Also noted is evidence of previous anterior cervical discectomy and fusion at C6–7 (dotted arrow). b MRI thoracic spine showing multi-level cord compression secondary to ossification of the ligamentum flavum (solid arrows). c Post-operative MRI thoracic spine several months after thoracic laminectomy showing large CSF pseudomeningocele (solid arrow). d MRI cervical and thoracic spine after pseudomeningocele repair surgery showing significant reduction in size (solid arrow). Also noted is the continued progression of the cervical ossification of the posterior longitudinal ligament with now obvious (asymptomatic) cord compression (dotted arrow) (GIF 263 kb)

High resolution image. (TIFF 420 kb)

Suppl. Fig. 4 Change in eGFR with age (JPEG 48 kb)

High resolution image (TIFF 1317 kb)

## References

[CR1] Addison WN, Nakano Y, Loisel T, Crine P, McKee MD (2008). MEPE-ASARM peptides control extracellular matrix mineralization by binding to hydroxyapatite: an inhibition regulated by PHEX cleavage of ASARM. J Bone Miner Res.

[CR2] Batra P, Tejani Z, Mars M (2006). X-linked hypophosphatemia: dental and histologic findings. J Can Dent Assoc.

[CR3] Beck-Nielsen SS, Brock-Jacobsen B, Gram J, Brixen K, Jensen TK (2009). Incidence and prevalence of nutritional and hereditary rickets in southern Denmark. Eur J Endocrinol.

[CR4] Beck-Nielsen SS, Brusgaard K, Rasmussen LM, Brixen K, Brock-Jacobsen B, Poulsen MR, Vestergaard P, Ralston SH, Albagha OM, Poulsen S, Haubek D, Gjorup H, Hintze H, Andersen MG, Heickendorff L, Hjelmborg J, Gram J (2010). Phenotype presentation of hypophosphatemic rickets in adults. Calcif Tissue Int.

[CR5] Bergwitz C, Juppner H (2010). Regulation of phosphate homeostasis by PTH, vitamin D, and FGF23. Annu Rev Med.

[CR6] Carpenter TO, Imel EA, Holm IA, Jan de Beur SM, Insogna KL (2011). A clinician’s guide to X-linked hypophosphatemia. J Bone Miner Res.

[CR7] Carpenter TO, Imel EA, Ruppe MD, Weber TJ, Klausner MA, Wooddell MM, Kawakami T, Ito T, Zhang X, Humphrey J, Insogna KL, Peacock M (2014). Randomized trial of the anti-FGF23 antibody KRN23 in X-linked hypophosphatemia. J Clin Invest.

[CR8] Cartwright DW, Masel JP, Latham SC (1981). The lumbar spinal canal in hypophosphataemic vitamin D-resistant rickets. Aust NZ J Med.

[CR9] Che H, Roux C, Etcheto A, Rothenbuhler A, Kamenicky P, Linglart A, Briot K (2016). Impaired quality of life in adults with X-linked hypophosphatemia and skeletal symptoms. Eur J Endocrinol.

[CR10] Connor J, Olear EA, Insogna KL, Katz L, Baker S, Kaur R, Simpson CA, Sterpka J, Dubrow R, Zhang JH, Carpenter TO (2015). Conventional therapy in adults with X-linked hypophosphatemia: effects on enthesopathy and dental disease. J Clin Endocrinol Metab.

[CR11] Coyac BR, Hoac B, Chafey P, Falgayrac G, Slimani L, Rowe PS, Penel G, Linglart A, McKee MD, Chaussain C, Bardet C (2017) Defective mineralization in X-linked hypophosphatemia dental pulp cell cultures. J Dent Res. 10.1177/0022034517728497 10.1177/0022034517728497 PMC6429567 28880715

[CR12] Delanaye P, Schaeffner E, Ebert N, Cavalier E, Mariat C, Krzesinski JM, Moranne O (2012). Normal reference values for glomerular filtration rate: what do we really know?. Nephrol Dial Transplant.

[CR13] Dunlop DJ, Stirling AJ (1996). Thoracic spinal cord compression caused by hypophosphataemic rickets: a case report and review of the world literature. Eur Spine J.

[CR14] Evans GA, Arulanantham K, Gage JR (1980). Primary hypophosphatemic rickets. Effect of oral phosphate and vitamin D on growth and surgical treatment. J Bone Joint Surg Am.

[CR15] Forestier-Zhang L, Watts L, Turner A, Teare H, Kaye J, Barrett J, Cooper C, Eastell R, Wordsworth P, Javaid MK, Pinedo-Villanueva R (2016). Health-related quality of life and a cost-utility simulation of adults in the UK with osteogenesis imperfecta, X-linked hypophosphatemia and fibrous dysplasia. Orphanet J Rare Dis.

[CR16] Gaucher C, Walrant-Debray O, Nguyen TM, Esterle L, Garabedian M, Jehan F (2009). PHEX analysis in 118 pedigrees reveals new genetic clues in hypophosphatemic rickets. Hum Genet.

[CR17] Hirao Y, Chikuda H, Oshima Y, Matsubayashi Y, Tanaka S (2016). Extensive ossification of the paraspinal ligaments in a patient with vitamin D-resistant rickets: case report with literature review. Int J Surg Case Rep.

[CR18] Horn A, Wright J, Bockenhauer D, Van’t Hoff W, Eastwood DM (2017). The orthopaedic management of lower limb deformity in hypophosphataemic rickets. J Child Orthop.

[CR19] Imel EA, Zhang X, Ruppe MD, Weber TJ, Klausner MA, Ito T, Vergeire M, Humphrey JS, Glorieux FH, Portale AA, Insogna K, Peacock M, Carpenter TO (2015). Prolonged correction of serum phosphorus in adults with X-linked hypophosphatemia using monthly doses of KRN23. J Clin Endocrinol Metab.

[CR20] Jacobs E, Martinez ME, Buckmeier J, Lance P, May M, Jurutka P (2011). Circulating fibroblast growth factor-23 is associated with increased risk for metachronous colorectal adenoma. J Carcinog.

[CR21] Kawaguchi A, Miyamoto K, Wakahara K, Hosoe H, Miura A, Hanamoto T, Shimizu K (2009). Surgical treatment of multiple spinal canal stenoses associated with vitamin D-resistant rickets. J Clin Neurosci.

[CR22] Khan FA, Koff MF, Noiseux NO, Bernhardt KA, O’Byrne MM, Larson DR, Amrami KK, Kaufman KR (2008). Effect of local alignment on compartmental patterns of knee osteoarthritis. J Bone Joint Surg Am.

[CR23] Kumar P, Henikoff S, Ng PC (2009). Predicting the effects of coding non-synonymous variants on protein function using the SIFT algorithm. Nat Protoc.

[CR24] Lee BN, Jung HY, Chang HS, Hwang YC, Oh WM (2017). Dental management of patients with X-linked hypophosphatemia. Restor Dent Endod.

[CR25] Levey AS, Stevens LA, Schmid CH, Zhang YL, Castro AF, Feldman HI, Kusek JW, Eggers P, Van Lente F, Greene T, Coresh J, Ckd EPI (2009). A new equation to estimate glomerular filtration rate. Ann Intern Med.

[CR26] Makitie O, Doria A, Kooh SW, Cole WG, Daneman A, Sochett E (2003). Early treatment improves growth and biochemical and radiographic outcome in X-linked hypophosphatemic rickets. J Clin Endocrinol Metab.

[CR27] Mathew TH, Johnson DW, Jones GR, Group Australasian Creatinine Consensus Working (2007). Chronic kidney disease and automatic reporting of estimated glomerular filtration rate: revised recommendations. Med J Aust.

[CR28] Moody A (2012) Adult anthropometric measures, overweight and obesity, Chap. 10. The Health and Social Care Information Centre, Leeds

[CR29] O’Malley SP, Adams JE, Davies M, Ramsden RT (1988). The petrous temporal bone and deafness in X-linked hypophosphataemic osteomalacia. Clin Radiol.

[CR30] O’Malley S, Ramsden RT, Latif A, Kane R, Davies M (1985). Electrocochleographic changes in the hearing loss associated with X-linked hypophosphataemic osteomalacia. Acta Otolaryngol.

[CR31] Pavone V, Testa G, Gioitta Iachino S, Evola FR, Avondo S, Sessa G (2015). Hypophosphatemic rickets: etiology, clinical features and treatment. Eur J Orthop Surg Traumatol.

[CR32] Quinlan C, Guegan K, Offiah A, Neill RO, Hiorns MP, Ellard S, Bockenhauer D, Hoff WV, Waters AM (2012). Growth in PHEX-associated X-linked hypophosphatemic rickets: the importance of early treatment. Pediatr Nephrol.

[CR33] Rabbani A, Rahmani P, Ziaee V, Ghodoosi S (2012). Dental problems in hypophosphatemic rickets, a cross sectional study. Iran J Pediatr.

[CR34] R-Core, Team (2016). R: a language and environment for statistical computing.

[CR35] Reid IR, Hardy DC, Murphy WA, Teitelbaum SL, Bergfeld MA, Whyte MP (1989). X-linked hypophosphatemia: a clinical, biochemical, and histopathologic assessment of morbidity in adults. Medicine (Baltimore).

[CR36] Ribeiro TR, Costa FW, Soares EC, Williams JR, Fonteles CS (2015). Enamel and dentin mineralization in familial hypophosphatemic rickets: a micro-CT study. Dentomaxillofac Radiol.

[CR37] Riccio AR, Entezami P, Giuffrida A, Dowling J, Forrest G, German JW (2016). Minimally invasive surgical management of thoracic ossification of the ligamentum flavum associated with X-linked hypophosphatemia. World Neurosurg.

[CR38] Rowe PS (2012). Regulation of bone-renal mineral and energy metabolism: the PHEX, FGF23, DMP1, MEPE ASARM pathway. Crit Rev Eukaryot Gene Expr.

[CR39] Rowe PS, Goulding JN, Francis F, Oudet C, Econs MJ, Hanauer A, Lehrach H, Read AP, Mountford RC, Summerfield T, Weissenbach J, Fraser W, Drezner MK, Davies KE, O’Riordan JL (1996). The gene for X-linked hypophosphataemic rickets maps to a 200-300kb region in Xp22.1, and is located on a single YAC containing a putative vitamin D response element (VDRE). Hum Genet.

[CR40] Sabbagh Y, Jones AO, Tenenhouse HS (2000). PHEXdb, a locus-specific database for mutations causing X-linked hypophosphatemia. Hum Mutat.

[CR41] Schwarz JM, Cooper DN, Schuelke M, Seelow D (2014). MutationTaster2: mutation prediction for the deep-sequencing age. Nat Methods.

[CR42] Seow WK (1984). X-linked hypophosphataemic vitamin D-resistant rickets. Aust Dent J.

[CR43] Seow WK, Needleman HL, Holm IA (1995). Effect of familial hypophosphatemic rickets on dental development: a controlled, longitudinal study. Pediatr Dent.

[CR44] Soehle M, Casey AT (2002). Cervical spinal cord compression attributable to a calcified intervertebral disc in a patient with X-linked hypophosphatemic rickets: case report and review of the literature. Neurosurgery.

[CR45] Souza AP, Kobayashi TY, Lourenco Neto N, Silva SM, Machado MA, Oliveira TM (2013). Dental manifestations of patient with vitamin D-resistant rickets. J Appl Oral Sci.

[CR46] Stickler GB, Morgenstern BZ (1989). Hypophosphataemic rickets: final height and clinical symptoms in adults. Lancet.

[CR47] Velan GJ, Currier BL, Clarke BL, Yaszemski MJ (2001). Ossification of the posterior longitudinal ligament in vitamin D-resistant rickets: case report and review of the literature. Spine (Phila Pa 1976).

[CR48] Wick CC, Lin SJ, Yu H, Megerian CA, Zheng QY (2017). Treatment of ear and bone disease in the Phex mouse mutant with dietary supplementation. Am J Otolaryngol.

