# Supplementary figures and images for: Outcome of adult patients with X-linked hypophosphatemia caused by PHEX gene mutations
Source: J Inherit Metab Dis. 2018 Feb 19;41(5):865–76. doi: 10.1007/s10545-018-0147-6 (PMC6133187; doi:10.1007/s10545-018-0147-6)

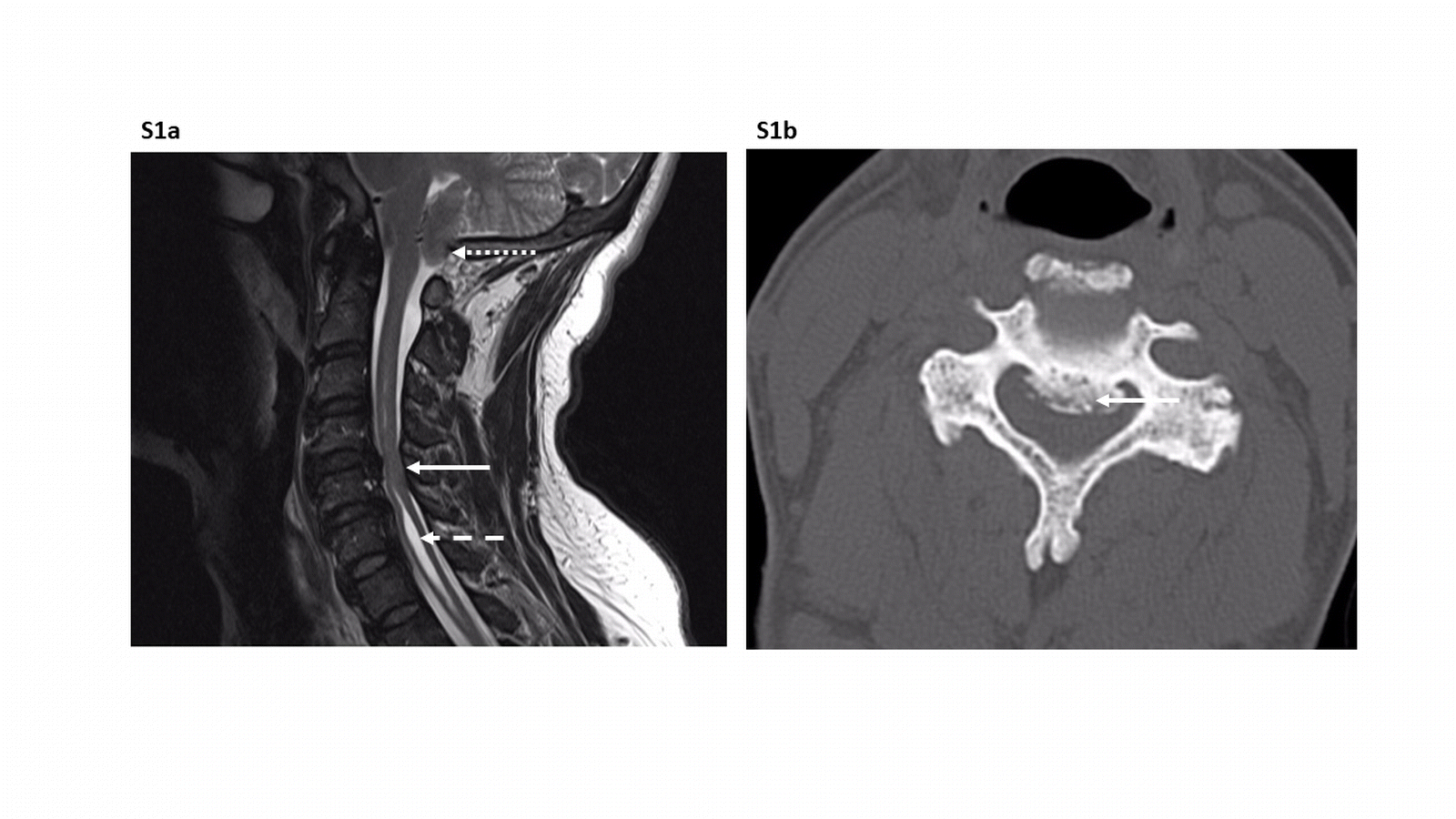

Supplement: Supplementary file 1 — a MRI cervical spine showing a Chiari malformation (dotted arrow), a syrinx at C6,C7 (dashed arrow) and spinal cord compression at C5–6 (solid arrow) secondary to ossification of the posterior spinal ligament. b CT cervical spine, an axial image showing significant reduction in the canal diameter due to ossification of the spinal ligament (solid arrow) (GIF 517 kb) [file 10545_2018_147_Fig4_ESM.gif]

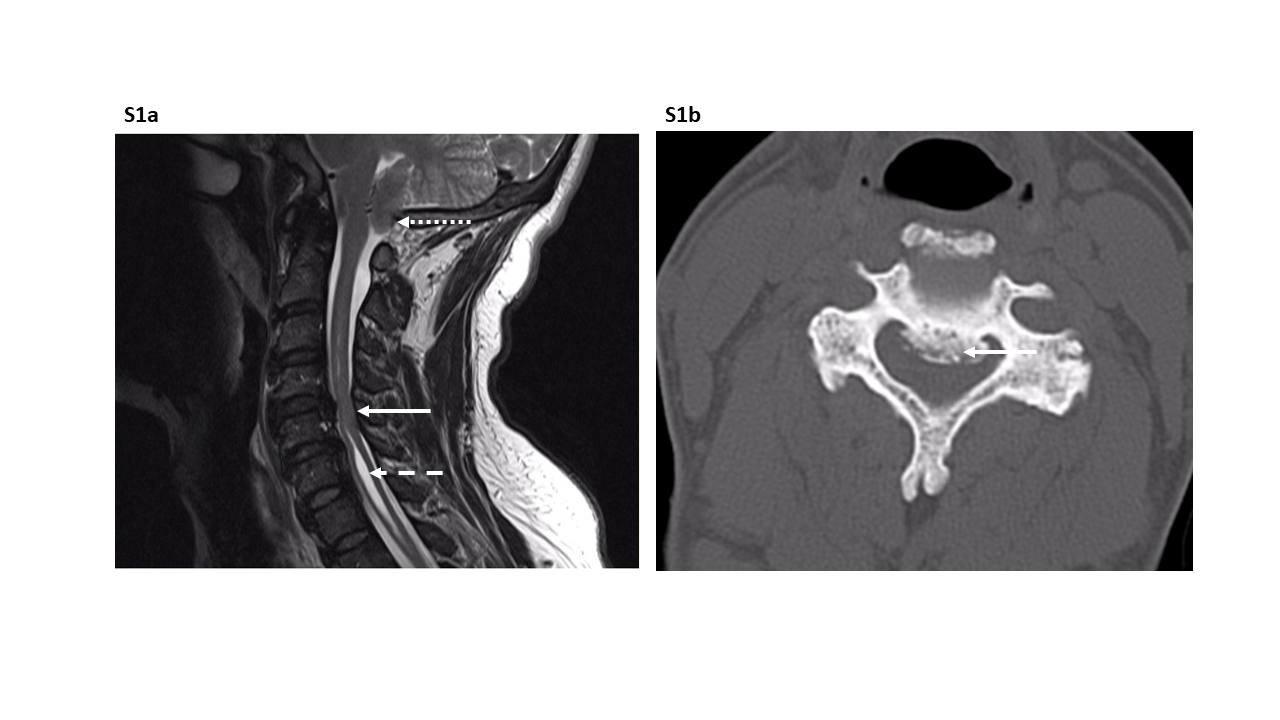

Supplement: Supplementary file 2 — High resolution image (TIFF 618 kb) [file 10545_2018_147_MOESM1_ESM.tif]

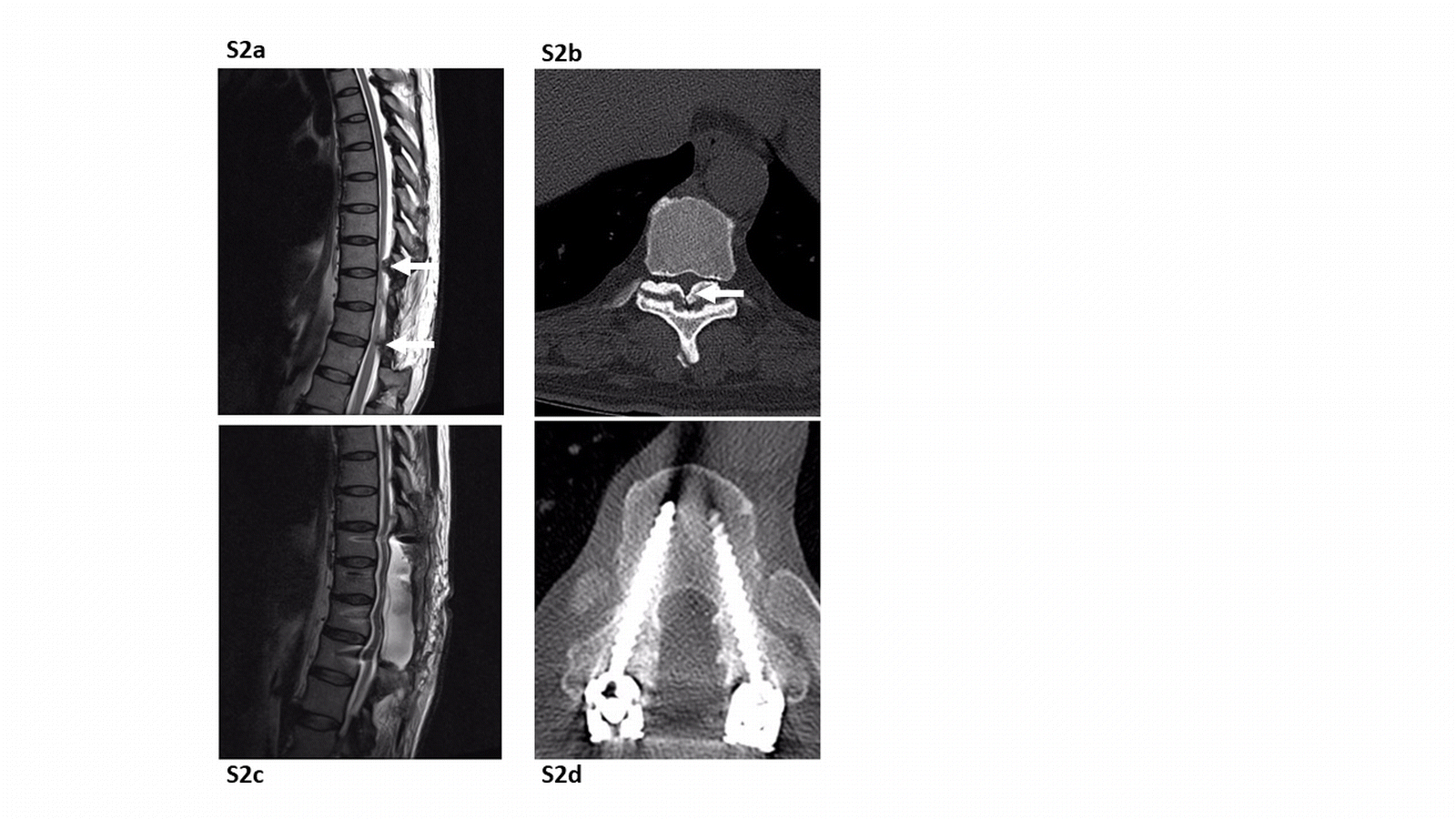

Supplement: Supplementary file 3 — a MRI thoracic spine showing previous thoracic laminectomy and new spinal cord compression above and below the decompression secondary to ossification of the ligamentum flavum. b CT thoracic spine axial image showing ossification of the ligamentum flavum causing canal stenosis. c Post-operative MRI thoracic spine showing well-decompressed spinal cord after thoracic laminectomy and fixation (the wavy cord appearance is due to metal artefact). d Post-operative CT thoracic spine showing absence/removal of the ossified ligamentum flavum after laminectomy and screw fixation (GIF 459 kb) [file 10545_2018_147_Fig5_ESM.gif]

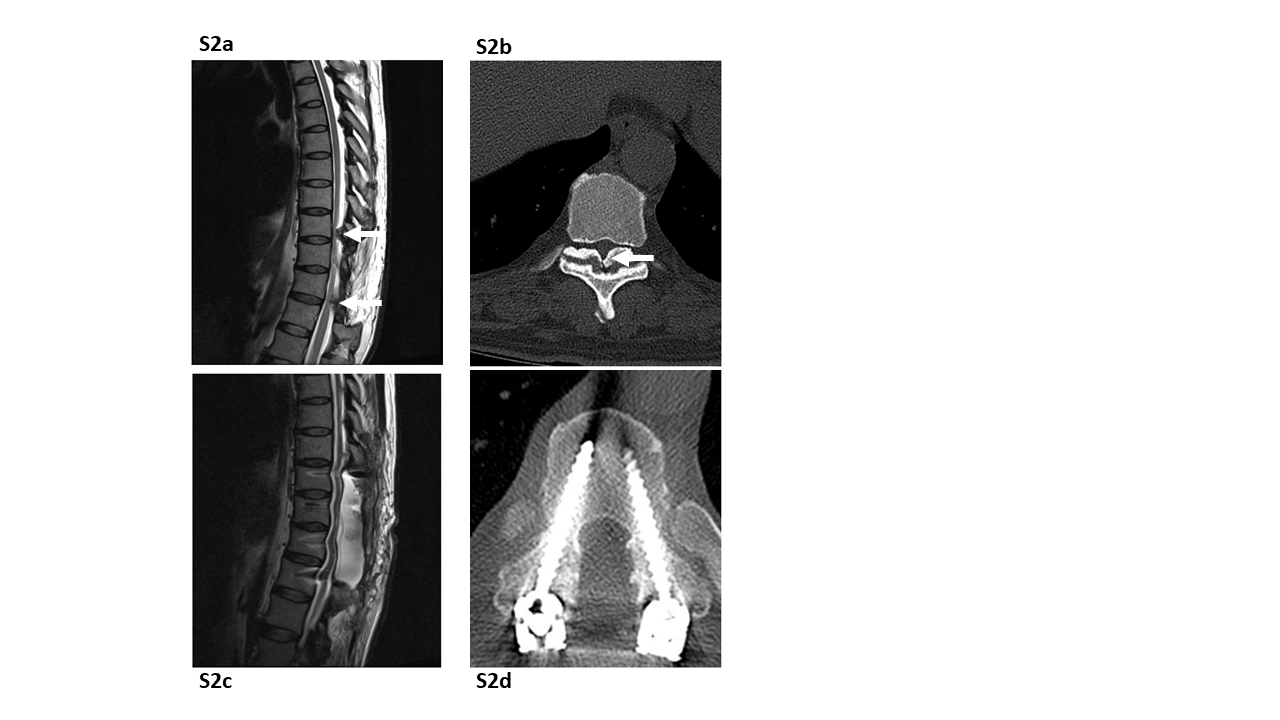

Supplement: Supplementary file 4 — High resolution image (TIFF 549 kb) [file 10545_2018_147_MOESM2_ESM.tif]

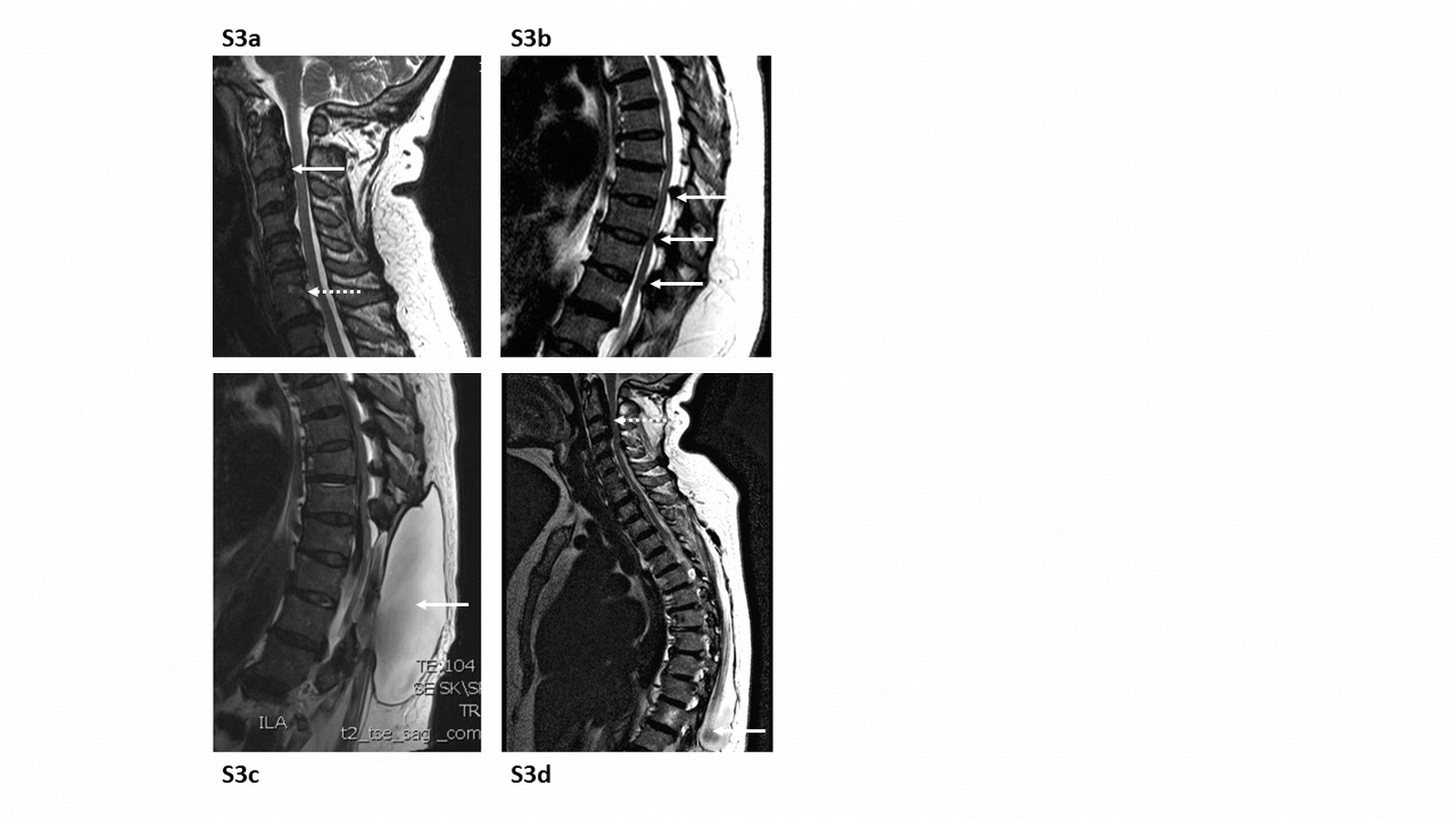

Supplement: Supplementary file 5 — a MRI cervical spine showing ossification of the posterior longitudinal ligament (solid arrow) causing minor canal stenosis. Also noted is evidence of previous anterior cervical discectomy and fusion at C6–7 (dotted arrow). b MRI thoracic spine showing multi-level cord compression secondary to ossification of the ligamentum flavum (solid arrows). c Post-operative MRI thoracic spine several months after thoracic laminectomy showing large CSF pseudomeningocele (solid arrow). d MRI cervical and thoracic spine after pseudomeningocele repair surgery showing significant reduction in size (solid arrow). Also noted is the continued progression of the cervical ossification of the posterior longitudinal ligament with now obvious (asymptomatic) cord compression (dotted arrow) (GIF 263 kb) [file 10545_2018_147_Fig6_ESM.gif]

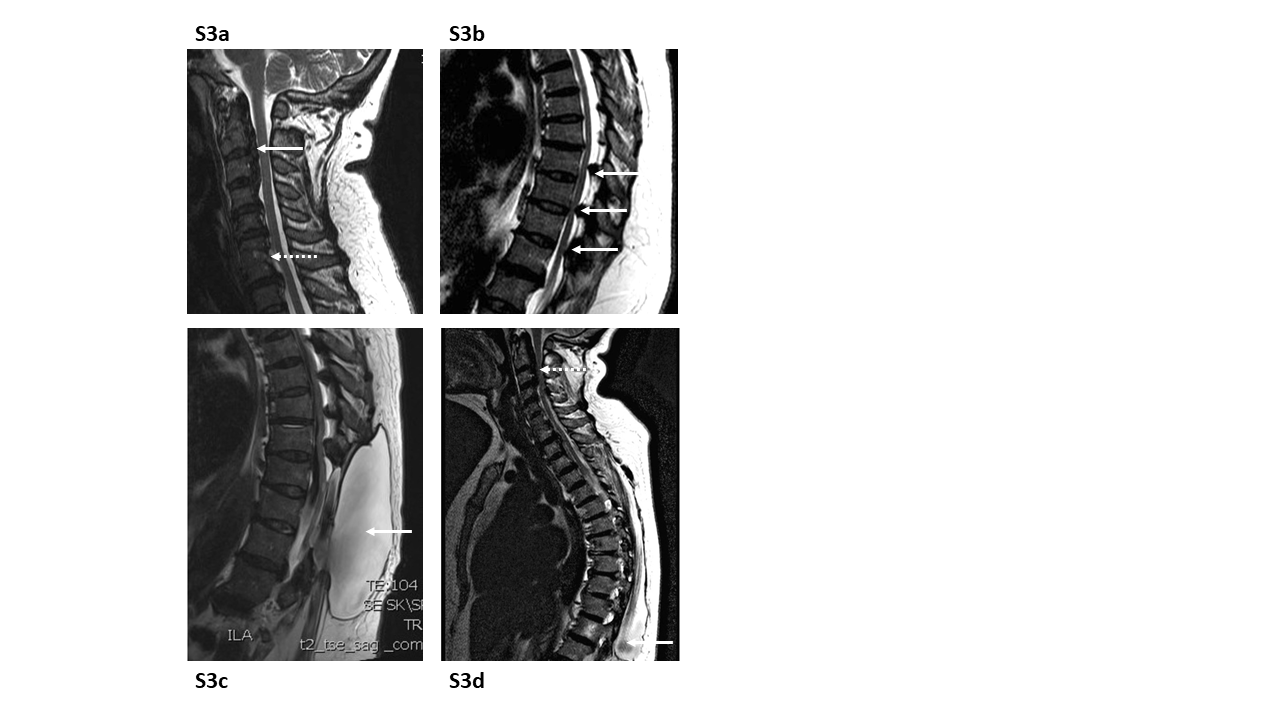

Supplement: Supplementary file 6 — High resolution image. (TIFF 420 kb) [file 10545_2018_147_MOESM3_ESM.tif]

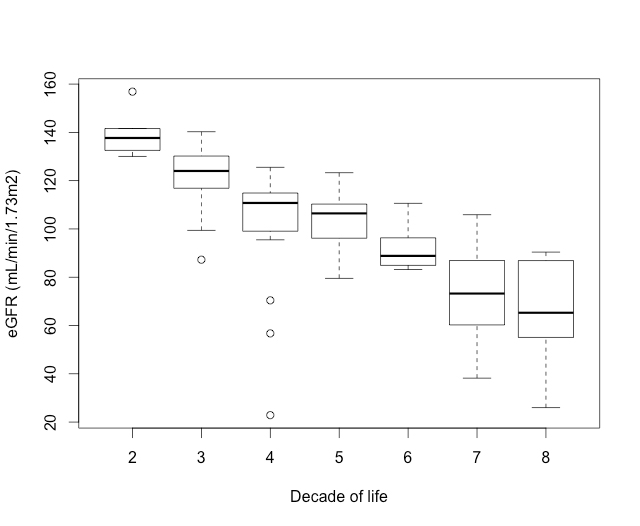

Supplement: Supplementary file 7 — Change in eGFR with age (JPEG 48 kb) [file 10545_2018_147_Fig7_ESM.jpg]

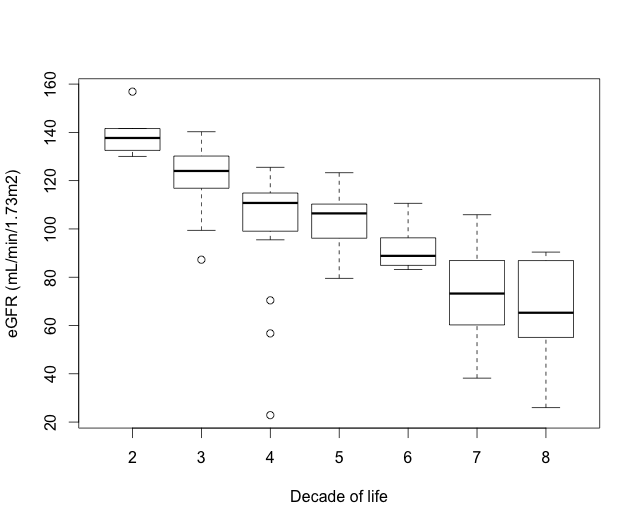

Supplement: Supplementary file 8 — High resolution image (TIFF 1317 kb) [file 10545_2018_147_MOESM4_ESM.tiff]
